# Real and predicted mortality under health spending constraints in Italy: a time trend analysis through artificial neural networks

**DOI:** 10.1186/s12913-018-3473-3

**Published:** 2018-08-29

**Authors:** Davide Golinelli, Andrea Bucci, Fabrizio Toscano, Filippo Filicori, Maria Pia Fantini

**Affiliations:** 1 Department of Biomedical and Neuromotor Sciences, Alma Mater Studiorum, University of Bologna, Bologna, Italy; 2 Department of Economics and Social Sciences, Marche Polytechnic University, Ancona, Italy; 3 Department of Healthcare Policy and Research, Weill Cornell Medical College, New York, USA; 4 The Oregon Clinic, Division of Minimally Invasive Gastrointestinal Surgery, Portland, OR USA

**Keywords:** Health expenditures, Mortality rate, Time trend analysis, Neural network models

## Abstract

**Background:**

After 2008 global economic crisis, Italian governments progressively reduced public healthcare financing. Describing the time trend of health outcomes and health expenditure may be helpful for policy makers during the resources’ allocation decision making process. The aim of this paper is to analyze the trend of mortality and health spending in Italy and to investigate their correlation in consideration of the funding constraints experienced by the Italian national health system (SSN).

**Methods:**

We conducted a 20-year time-series study. Secondary data has been extracted from a national, institution based and publicly accessible retrospective database periodically released by the Italian Institute of Statistics. Age standardized all-cause mortality rate (MR) and health spending (Directly Provided Services - DPS, Agreed-Upon Services - TAUS, and private expenditure) were reviewed. Time trend analysis (1995–2014) through OLS and Multilayer Feed-forward Neural Networks (MFNN) models to forecast mortality and spending trend was performed. The association between healthcare expenditure and MR was analyzed through a fixed effect regression model. We then repeated MFNN time trend forecasting analyses on mortality by adding the spending item resulted significantly related with MR in the fixed effect analyses.

**Results:**

DPS and TAUS decreased since 2011. There was a mismatch in mortality rates between real and predicted values. DPS resulted significantly associated to mortality (*p* < 0.05). In repeated mortality forecasting analysis, predicted MR was found to be lower when considering the pre-constraints health spending trend.

**Conclusions:**

Between 2011 and 2014, Italian public health spending items showed a reduction when compared to prior years. Spending on services directly provided free of charge appears to be the financial driving force of the Italian public health system. The overall mortality was found to be higher than the predicted trend and this scenario may be partially attributable to the healthcare funding constraints experienced by the SSN.

**Electronic supplementary material:**

The online version of this article (10.1186/s12913-018-3473-3) contains supplementary material, which is available to authorized users.

## Background

The social and health effects of economic downturns have been widely analyzed in the past years, particularly after the 2008 global crisis [1–4]. Recently, Watkins et al. sought to determine whether public spending on health and social care has affected England population health status and more specifically mortality rates [5]. The authors concluded that NHS spending constraints since 2010 might have been associated with a substantial mortality gap in England, thus underlining the association between spending on health and health outcomes.

Over the past years, similar public expenditure constraints have been experienced by many European countries, whose national governments have performed cuts and reallocations on healthcare and social spending, as a consequence of 2008 economic crisis [6–8]. Similar policies for spending control have been adopted by countries with single-payer healthcare systems [2, 9–11]. Among these, Italy, with a 60 million population universally covered by the National Health Service (Servizio Sanitario Nazionale, SSN) represents an exemplifying case to be analyzed.

Eurostat reports that social protection expenditure in the European Union (EU) has increased slightly, from 28.6% of GDP in 2010 to 29.0% in 2015. Social protection expenditure in Italy was 30.0% of GDP in 2015 [12]. When compared to his European partners Italy presents proportionally lower public spending on “sickness/healthcare and disability” (28.9% versus 37.3% of EU mean, reported as % of total social benefits) [12]. Despite this, Italy maintains high levels of performance in terms of population health status [13], following the trend of the last century of Italian history. Particularly, Italian mortality rate presented a consistent decline from 1901 to 2008, as reported in Vercelli et al., with an annual percent change of − 3.2% and − 3.9% respectively in males and females aged 0–49 years, and of − 0.9% and − 1.7% respectively in males and females aged 50–69 [14]. This trend is comparable with other high-income countries [15].

Over the last years Italian governments progressively reduced public healthcare financing threatening the future sustainability of the SSN. Moreover, Italy has been struggling with rising public debt and a stagnant growth rate of its overall economy [14]. Specifically, Italian healthcare expenditure grew by 7.5% yearly from 2001 to 2005, by 3.1% yearly from 2006 to 2010 and decreased by 0.1% yearly from 2011 to 2015 [15, 16]. All of this is happening in a context of growing healthcare needs and costs, given the aging of the population (globally Italy has the third highest proportion of people aged over 75 years) [17–19] and the development of increasingly expensive innovative treatments and technologies.

Describing the time trend of health outcomes and health expenditure, and investigating their potential association may be helpful for policy makers during the resources’ allocation/reallocation decision making process. While conventional time trend and forecast analyses have been used and validated in several studies [20–22], Artificial Neural Networks (ANN) model is still a novel technique. However, many recent studies have demonstrated that Neural Networks models provide more accurate prediction of clinical outcomes in comparison to conventional methods [23–25]. To the best of our knowledge, the present study introduces ANN models in health services research. No previously published studies indeed have analyzed the time trend of health funding and outcomes, and particularly the impact of the health spending constraints on the overall health status of the Italian population, through ANN models.

The concept behind this study is that the trend of Italian health expenditure - in particular its progressive reduction after year 2010 [14, 15] - has potentially affected Italian population mortality rate. The aim of this study is therefore to analyze the time trend of mortality rate and health spending in Italy and to investigate their correlation in consideration of the funding constraints experienced by the publicly financed Italian national health system.

## Methods

### Conceptual framework and study design

We conducted a time-series study with 20-year time trend analyses (1995–2014) using Ordinary Least Squares and Feed-forward Neural Networks models to forecast mortality and health spending trend. In order to validate our model and to implement our approach we also used life expectancy at birth as alternative health outcome. We made this choice because life expectancy has been widely adopted in literature as a comparison indicator of population overall health status and as a performance indicator of national healthcare systems [5]. The association between the three main items of Italian healthcare expenditure (i.e. public, semi-private or agreed-upon, and private health spending) and overall mortality rate through a 20-year panel data analysis was quantified. We again forecasted mortality rate by adding real and predicted values of health spending to the model.

In summary, our analysis includes the following steps: 1) Confirming the presence of a break year by identifying the first year health spending constraints started, through a descriptive analysis. 2) Performing time trend and forecast analyses on mortality rate and health expenditure items including the time periods pre−/post- health spending break year. 3) Investigating the correlation between health spending and mortality. 4) Performing time trend and forecast analyses on mortality rate including the expenditure items associated with mortality rates.

### Data

Secondary data on health expenditure, mortality, life expectancy and socio-demographic characteristics was extracted from *Health for All*, a publicly accessible retrospective database periodically released by the Italian National Institute of Statistics (ISTAT), based on a World Health Organization platform [26]. Data was analyzed differently in time trend analysis and fixed effects regression. For the time trend analysis, we relied on a time series dataset, where mortality rate, health expenditure items and control variables were aggregated at national level over a period of 20 years, from 1995 to 2014. The fixed effects regression analysis, instead, has been based on a panel database across the 20 Italian regions over a period of 20 years, from 1995 to 2014.

### Population mortality data

Age-standardized all-cause mortality rate (male + female deaths per 10,000 population, MR) was used as the endpoint of our study and as a measure of population health status.

### Spending and resources data

ISTAT expenditure data aggregation relies on definitions reported in the OECD manual “A System of Health Accounts”, which provides a comparable description of the financial flows related to the consumption of healthcare goods and services [27].

The three main expenditure items which constitute the total national health spending, as provided by ISTAT, were taken into account (per capita and inflation-adjusted) [28]: public, semi-private or agreed-upon (i.e. all publicly funded but privately provided services) and private health spending (Additional file 1). Italian public health expenditure is defined as the sum of directly provided services (DPS) and services funded by the Italian National Healthcare Service (SSN) but supplied by private healthcare providers, Total Agreed-Upon Services (TAUS). TAUS includes several expenditure items, such as expenditure for outpatient prescription drugs, general practitioners care, outpatient specialist medical care, privately delivered hospital care and psychiatric support and rehabilitation. Family healthcare expenditure (FHE) was included amongst the variables in the analysis to capture private/out-of-pocket spending on health. Publicly funded health expenditure (i.e. DPS + TAUS) accounted for 75% of total health spending in Italy in 2016. In the same year, direct private spending (FHE in our database) accounted for 23% [29]. In stark contrast with other European countries that present a consistent share of population with mixed profile of health care coverage (e.g. in Spain 12.45% of the population had mixed health care coverage in 2011–12 [30]), the intermediate private health care coverage (i.e. private insurances) proportion in Italy is very low (2% in 2016). Unfortunately, specific data with regional detail on private insurances were not available and therefore not included in our study.

Items of “social protection expenditure” in Italy are mainly attributable to spending on retirement benefits [12, 31] and were therefore not included. The rest of social protection services in Italy is covered by family private spending (FHE) and by the SSN (e.g. rehabilitation services and home cares for fragile and/or terminal patients), which are included in DPS and TAUS [29, 32].

### Statistical analysis

#### Time trend analysis

To enhance the accuracy of our approach, we used two techniques for time trend analysis: Ordinary Least Squares (OLS) and Multilayer Feed-forward Neural Network (MFNN). These models have been proved to be accurate in predicting health outcomes but, to the best of our knowledge, they have never been implemented to analyze the time trend of health funding and mortality rate in a similar setting [23–25]. Time trend analyses were conducted using OLS and MFNN models with MR as dependent variable trended by calendar year starting from 1995. We then performed subgroup analysis with each expenditure item (DPS, TAUS and FHE). Analyses were repeated using male and female life expectancy as alternative health outcome to assess for potential worsening of the population health status.

### Multilayer feed-forward neural networks model

Time trend analysis and forecasts were also performed through an artificial neural network model. A neural network model can be seen as a network of neurons organized in three layers: the input layer, the intermediate layer, which contains hidden nodes, and the output layer. In comparison with linear regression models (such as OLS), the presence of an intermediate layer provides non-linear analysis through the network described in Additional file 2. In this model, known as Multilayer Feed-forward Neural Network, the coefficients or “weights” are obtained from a “learning algorithm” that minimizes a “cost” function as the mean squared error. In this framework, the information moves in a single direction (forward), from the input layer through the hidden/intermediate layer to the output layer. The outputs from the nodes in one layer are combined through a weighted linear combination and used as inputs for the following layer. A more detailed explanation of the model is reported in the Additional file 3 (“Section Mathematics/formula”) of the Additional files.

The neural network approach is preferred to linear regression models when the number of observations is relatively small, as in our case [24, 25]. Furthermore, the presence of the intermediate layer introduces non-linearity in the model that can better capture the effect of outliers.

### Fixed effect regression analyses

Fixed effect regression models were used to investigate the association between health expenditure and MR, using the panel data across the 20 Italian Regions. We first determined whether a lag effect between health spending and mortality was present, by adding 0, 1 and 2 year lags to the model (see Additional file 4).

After excluding the lag effect, we created 5 different models, by including control variables considered important health determinants (expenditure and control variables are described in Additional file 1). The 5 models analyzed were:

- Expenditure model:
    $$ {MR}_{i,t}={\beta}_0+{\beta}_1{DPS}_{i,t}+{\beta}_2{TAUS}_{i,t}+{\beta}_3{FHE}_{i,t}+\gamma t+{\alpha}_i\kern0.5em +{\varepsilon}_{i,t} $$
  Where *β*_0_ is the constant term, θ = {β_1_, β_2_, β_3_} is the vector of coefficients of health expenditure items (*DPS*, *TAUS* and *FHE*), *α*_*i*_ is a vector of Regional fixed effects and *γ* is the coefficient on the time trend *t*.
- Social model:
    $$ {MR}_{i,t}={\beta}_0+{\beta}_1{DPS}_{i,t}+{\beta}_2{TAUS}_{i,t}+{\beta}_3{FHE}_{i,t}+\delta {Soc}_{i,t}+\kern0.5em \gamma t+{\alpha}_i\kern0.5em +{\varepsilon}_{i,t} $$
  Where *δ* is the vector of coefficients of the social condition control variables (Unemployment, Educational level and GDP).
- Lifestyle model:
    $$ {MR}_{i,t}={\beta}_0+{\beta}_1{DPS}_{i,t}+{\beta}_2{TAUS}_{i,t}+{\beta}_3{FHE}_{i,t}+\tau {Life}_{i,t}\kern0.5em +\gamma t+{\alpha}_i\kern0.5em +{\varepsilon}_{i,t} $$
  Where *τ* is the vector of coefficients related to lifestyle control variables (Smoke rate, Obesity rate and Sport active people).
- Healthcare model:
    $$ {MR}_{i,t}={\beta}_0+{\beta}_1{DPS}_{i,t}+{\beta}_2{TAUS}_{i,t}+{\beta}_3{FHE}_{i,t}+\lambda {Health}_{i,t}\kern0.5em +\gamma t+{\alpha}_i\kern0.5em +{\varepsilon}_{i,t} $$
  Where is the vector of coefficients associated to healthcare control variables (Number of Hospital Beds and Number of Nurses).
- Overall model:
    $$ {MR}_{i,t}\ {\beta}_0+{\beta}_1{DPS}_{i,t}+{\beta}_2{TAUS}_{i,t}+{\beta}_3{FHE}_{i,t}+\delta {Soc}_{i,t}+\tau {Life}_{i,t}+\lambda {Health}_{i,t}\kern0.5em +\gamma t+{\alpha}_i\kern0.5em +{\varepsilon}_{i,t} $$
  Where all the control variables have been included.

A time constant and a time trend were included in each model to approximate the time trend analyzed in the previous sections [33–36].

The fixed effect regression model is described in the Additional file 3 (“Section Mathematics/formula”) of the Additional files. The regression model uses ecological-level data only. One potential risk in using ecological measures is the ‘ecological fallacy’. However, because our analysis only uses the Region itself as the unit of analysis and does not make generalizations about individuals or specific population groups within each Region, it does not risk an ecological fallacy [37].

### Mortality forecast analyses

On the basis of the results obtained from fixed effect analyses (Table 2 and Additional file 4), we repeated MFNN time trend forecasting analyses on mortality rate by adding the spending item resulted significantly related with MR in all models (i.e. DPS). Due to the linear nature of the predicted DPS, when using predicted DPS the OLS model presents a collinearity problem (i.e. predicted DPS and time trend correlate as regressors). We therefore decided to rely on the MFNN model to forecast mortality rate through predicted health spending.

**Fig. 1:**
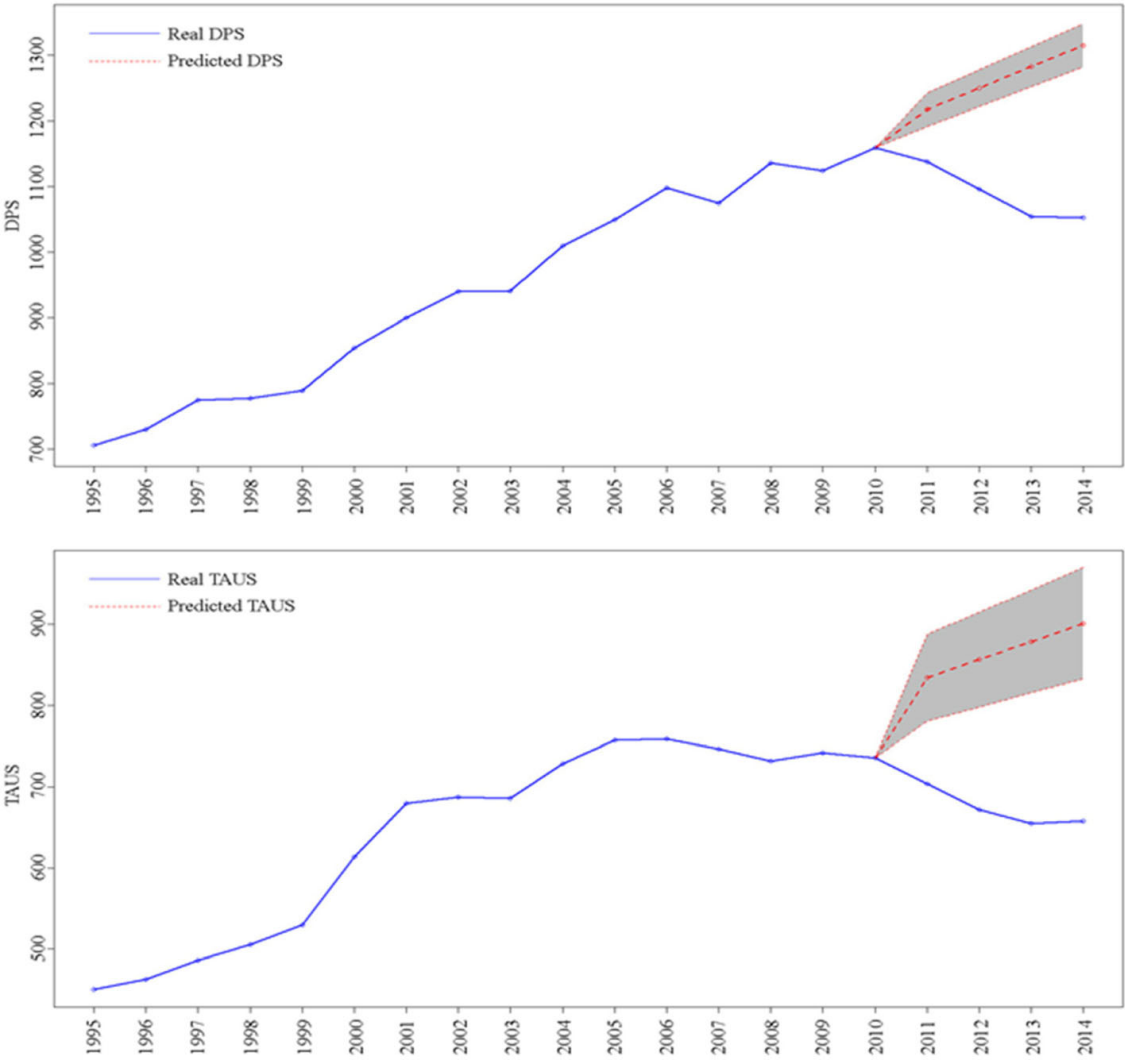
Time trend of DPS and TAUS using OLS analysis. Blue line is real DPS trend. Red dots line is predicted DPS trend. The 95% CIs are denoted by the grey-coloured area

In particular, we performed the analyses with MR as dependent variable and DPS and calendar year as independent variables. We predicted mortality rate through two separate approaches. Firstly, real DPS values were used (i.e. DPS real data for the period 2011–2014). Secondly, we included the values of DPS predicted through OLS (i.e. out-of-sample DPS forecasts, reported in Table 1 and Fig. 1, namely “fitted” DPS). We included only values of DPS predicted by linear regression (OLS analysis), since we looked at a linear time trend.

**Table 1:** OLS and Neural Network time trend forecast analysis (2011–2014) of mortality rate (MR) and health spending items (DPS, TAUS, FHE). MR is in deaths/10.000. DPS, TAUS and FHE are in € per capita

Year	Real	OLS		Neural Network	
		Prediction	95% interval	Prediction	95% interval
MR					
2011	83.74	82.07	(80.15, 83.99)	84.25	(81.90, 86.60)
2012	84.78	80.06	(77.96, 82.15)	83.34	(80.92, 85.76)
2013	80.59	78.04	(75.77, 80.32)	82.79	(80.42, 85.17)
2014	78.29	76.03	(73.57, 78.49)	82.32	(79.93, 84.70)
DPS					
2011	1137.21	1216.77	(1190.78, 1242.75)	1153.13	(1124.99, 1181.27)
2012	1095.43	1249.18	(1220.81, 1277.56)	1170.69	(1138.38, 1203.01)
2013	1054.15	1281.60	(1250.78, 1312.41)	1171.92	(1138.19, 1205.65)
2014	1052.00	1314.01	(1280.72, 1347.31)	1180.51	(1147.86, 1213.16)
TAUS					
2011	703.79	834.01	(780.44, 887.57)	741.37	(720.93, 761.81)
2012	671.52	856.38	(797.89, 914.88)	742.48	(720.91, 764.05)
2013	654.96	878.76	(815.23, 942.29)	742.54	(721.67, 763.41)
2014	658.00	901.14	(832.49, 969.77)	742.39	(721.39, 763.39)
FHE					
2011	582.81	545.55	(508.79, 582.31)	502.61	(479.75, 525.47)
2012	560.45	548.48	(508.34, 588.62)	520.69	(487.36, 554.03)
2013	544.63	551.40	(507.81, 594.99)	512.96	(480.39, 545.52)
2014	553.00	554.33	(507.23, 601.43)	525.05	(488.70, 561.41)

The aim of this analysis was to obtain an estimate of the MR trend (2011–2014), relative to real and predicted health spending (real and predicted values of DPS, as reported in Table 1 and Fig. 1). The resulting model answers to the following question: what would have been the mortality values if public health spending had followed the trend preceding funding constraints?

For all statistical analysis we used R, version 3.4.3, R Development Core Team, © The R Foundation.

## Results

### Descriptive analysis

Descriptive analysis identified 2011 as the first year health spending constraints started. The average yearly increase in DPS was 3.40% from 1995 to 2010 (Additional file 5), alculated as an average of the yearly growth in the period 1995–2010. Between 2011 and 2014, the decline of DPS was − 1.45%. Spending on TAUS increased by 3.41% from 1995 to 2010 and decreased by 2.71% between 2011 and 2014. Spending on FHE increased by 1.45% from 1995 to 2010 and by 0.12% between 2011 and 2014. MR declined steadily by 2.04% between 1995 to 2010 and then by 1.69% between 2011 and 2014 (Additional file 5).

### Time trend analyses

**Fig. 2:**
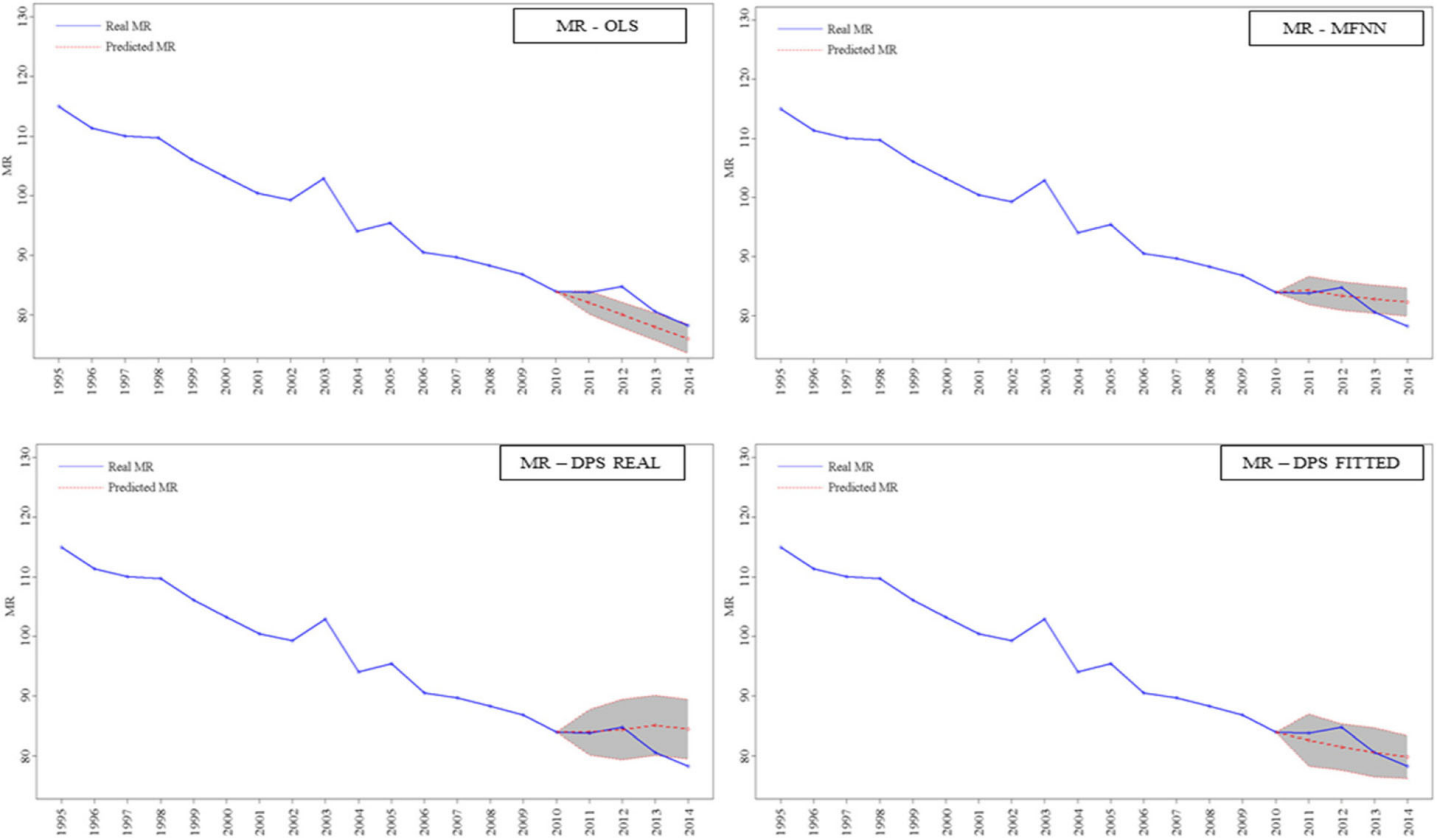
Time trend of MR using OLS (MR – OLS) and MFNN analysis (MR – MFNN) and repeated time trend forecast analysis of MR using Neural Network and adding real (MR – DPS REAL) and predicted (MR – DPS FITTED) DPS to the model. Blue line is real MR trend. Red dots line is predicted MR trend. The 95% CIs are denoted by the grey-coloured area

OLS time trend analysis on MR showed a gap between real and predicted values from 2011 to 2014 (Tables 1 and Fig. 2). As an example, in 2013 real MR was 80.59 (deaths/10,000), while the predicted values were 78.04 with OLS analysis. This suggests that if MR had followed the pre-2011 trend it would have been generally lower than real observed values. Repeated OLS analysis on life expectancy showed a trend similar to MR (Additional files 6, 7, 8). Predicted MR through MFNN showed different results particularly in years 2013 and 2014 where predicted MR resulted higher than the actual mortality rates.

Time trend analyses of health spending items with OLS analysis and MFNN models yielded similar results when using DPS and TAUS as dependent variables (Tables 1, Additional files 9, 10). Public spending on DPS and TAUS substantially decreased since 2011 and did not follow the trend from the prior years. As an example, in 2012 real values of DPS were 1095.43 € per capita, substantially differing from predicted values obtained through OLS (1249.18 € per capita) and MFNN (1170.69). In the same year FHE had predicted values closer to the real ones (Table 1 and Additional files 11, 12).

### Fixed effect regression models

The regression models are shown in Table 2. DPS displayed a statistically significant negative association with MR in all 5 models considered (*p* < 0.05). Regarding the other expenditure variables, TAUS was not found to be associated with MR and FHE showed a significant negative association with MR only in model b), where social condition was considered (per capita GDP, educational level and unemployment rate).

**Table 2:** Fixed effect regression models with MR as dependent variable

	Model a)	Model b)	Model c)	Model d)	Model e)
DPS	−0.0077** (0.0280)	−0.0061*** (0.0060)	−0.0111 *** (0.0007)	− 0.0099 *** (0.0013)	−0.0107*** (0.0047)
TAUS	−0.0019 (0.6855)	0.0001 (0.9801)	0.0033 (0.5172)	−0.0025 (0.6219)	0.0071 (0.1812)
FHE	−0.0049 (0.3618)	−0.0052** (0.0373)	−0.0023 (0.6952)	− 0.0041 (0.3259)	0.0018 (0.6190)
Social Condition					
Unemployment	–	0.0395 (0.5648)	–	–	0.0175 (0.8443)
Educational level	–	−0.0029 (0.9729)	–	–	−0.0652 (0.5908)
GDP	–	−0.0001 (0.2329)	–	–	−0.0002 (0.2949)
Lifestyle					
Smoke rate	–	–	−0.0295 (0.7451)	–	0.0232 (0.8346)
Obesity rate	–	–	−0.0499 (0.7843)	–	−0.0701 (0.7094)
Sport active people	–	–	−0.1316 (0.2334)	–	−0.1861* (0.0542)
Healthcare					
HB	–	–	–	−0.0629 (0.3192)	−0.2786** (0.0276)
NN	–	–	–	0.0006** (0.0388)	0.0004 (0.1775)

*** = *p* < 0.01; ** = *p* < 0.05; * = *p* < 0.1. Control variables are described in Additional file 1

### Mortality forecast

After assessing the association between DPS and MR, we repeated time trend analyses with MR as the dependent variable using DPS and calendar year as independent variables. MR values were substantially lower when predicted DPS (“fitted” DPS) was used compared to real DPS (Table 3 and Fig. 2) and to MR values obtained without the inclusion of health spending to the model (Table 1 and Fig. 2). When fitted DPS was used as regressor, predicted MR was 80.57 deaths/10,000 in 2013, substantially lower than MR predicted with real DPS (85.06 deaths/10,000) and MR predicted without considering health spending effect (82.79 deaths/10,000). The analysis further underlines the effect of DPS on mortality rate, which smooth and generally reduce mortality predicted values.

**Table 3:** Estimation results of MR adding DPS real and predicted (fitted) values to the model. MR is in deaths/10.000

Year	Real	Real values		Fitted values	
		Prediction	95% interval	Prediction	95% interval
Forecasts with Neural Network					
2011	83.74	83.95	(80.16, 87.71)	82.61	(78.25, 86.97)
2012	84.78	84.38	(79.30, 89.47)	81.49	(77.65, 85.35)
2013	80.59	85.06	(80.06, 90.06)	80.57	(76.45, 84.69)
2014	78.29	84.45	(79.46, 89.44)	79.81	(76.24, 83.38)

## Discussion

Our results highlight that, between 2011 and 2014, the two main items of Italian public health spending (DPS and TAUS) decreased and actually become negative when compared to the 1995–2010 trend. The growth of private health spending still remains positive in the period 2011–2015, but the growth is significantly reduced. Lastly, the Italian mortality rate showed a steeper decline between 1995 and 2010 than between 2011 and 2014, thus underlining a potential worsening of the population health status. This assumption is confirmed by the similar results yielded by life expectancy.

Time trend analyses confirmed that the health spending reduction experienced by the Italian system started in year 2011 and mainly involved the items of public health spending (public spending on directly provided and agreed-upon services). This can be attributable to the inevitable effects of the 2008 global economic crisis, but also to legitimate provisions of the Italian national and regional governments in terms of funding cuts or reallocation [9]. On the other hand private health expenditure showed a real and predicted trend which was hard to interpret. This is probably due to a number of factors influencing private spending on healthcare (ability to pay, individual education, etc.), as confirmed by fixed effect analysis, where FHE results significantly and inversely associated with mortality rate only when social condition is considered.

Time trend analyses on mortality rate showed the presence of a mismatch between real and predicted mortality. OLS analysis suggests that if MR had followed the pre-2011 trend it would have been generally lower than real observed values.

Mortality was differently predicted through MFNN models. Neural networks allow non-linear forecasted time series and this may produce different forecasts from linear regression models. The neural network forecasting accuracy appears limited when the regressors include uniquely a lag of the dependent variable and a time trend [29], thus the accuracy of the model might be further enhanced by adding at least an exogenous regressor (other than the time trend), as conducted in the repeated analyses with health spending as additional independent variable. Similarly to other studies [23–25, 38], neural networks models seem to provide accurate prediction of health outcomes, as compared with traditional methods, particularly when at least an exogenous variable is considered.

The panel data analysis described the association between health spending and mortality and showed that DPS is the only spending item significantly related with mortality in all models considered. This result is consistent with our prior findings [39]. Our models confirm that DPS is the financial driving force of the publicly funded Italian health system, and a potential determinant of the Italian population health status.

When we repeated the MFNN time trend analyses of mortality rate with health spending as additional independent variable, MR values obtained with predicted DPS resulted substantially lower than MR values obtained with real DPS and MR predicted only by time trend. This finding underscores that if DPS spending had hypothetically followed the pre-2011 trend, Italian mortality rate during the forecasted years could have been lower. The analysis further underlines the effect of DPS on mortality rate, which smooth and generally reduce mortality predicted values.

Many authors reviewed the wide-ranging literature about the health effects of economic hardship, highlighting that findings were not consistent [1, 40]. Our analysis identified a structural break of public health spending and mortality rate time series in year 2011 and demonstrated a counter-cyclical relationship between health spending and population overall health status, confirming the results of previous studies [3, 10]. In the Italian context, private direct health spending (FHE) presented a stable trend during the period of economic crisis and was not associated with mortality rate. Public spending on health (DPS), instead, reduced during the same period and resulted significantly related with mortality rate.

Our study therefore gives some insights into the association between health expenditure and population health status suggesting that the health spending constraints experienced by the Italian public health system may have partially influenced mortality rate. This evidences call for a reflection. As stated by Stuckler et al., health spending cannot be considered as the only determinant of population mortality [7]. Nonetheless, policy makers should be aware that different health spending items may have different impact on population health status and should consider the potential effect of a reduced health spending on mortality rate. This is particularly important during the resources’ allocation/reallocation process.

### Strengths and weaknesses of the study

This paper aimed at analyzing the time trend of total Italian health spending divided in its main components - which allow for a more detailed approach - to understand the expenditure gap in the period 2011–2014. To the best of our knowledge, this is also the first study adopting innovative neural networks models to estimate mortality and health spending trend and to analyze healthcare outcomes with a focus on the years of economic constraint. Nonetheless further studies are needed to test and validate such technique in different time-series and forecast analyses.

Our study includes the analysis of data available for 6 years after 2008 global economic crisis (2009–2014). This allowed us to rely on a more consistent number of years if compared with the previous literature on the impact of the financial crisis on health outcomes in Europe [10].

However, we acknowledge that measuring health outcomes with MR may misestimate the effects of health spending on the overall population health status. Another limitation is that the analytic approach of this study may not support causal inference: contingent economic hardship may lead simultaneously to worse health outcomes and to reduced tax revenues, thus creating an apparent association between lower spending on health and mortality. In addition, the level of geographical aggregation could influence the association between expenditure and mortality. With respect to other similar studies [3, 5, 7] another limitation lies in the difficulty to obtain reliable data on pure social spending. However, in our study this spending component is partially included in other expenditure items analyzed.

### Future lines of research

A differences-in-differences approach with panel data at regional level would shed the light on the heterogeneity of the Italian context. Moreover, in order to deepen the analysis of the present study, a comparison across the Italian regions could be performed using the same methodology to highlight the differences across the regions.

## Conclusions

Our study confirms that, between 2011 and 2014, Italian public health spending items showed a reduction when compared to prior years. Spending on services directly provided free of charge appears to be the financial driving force of the Italian public health system. The overall mortality was found to be higher than the predicted trend and this scenario may be partially attributable to the healthcare funding constraints experienced by the Italian national health system.

## Additional files

Additional file 1: Spending and control variables. Variables used in time trend analysis and fixed effect regression models. (DOCX 14 kb)

Additional file 2: Multilayer Feed-forward Neural Networks model. A neural network model can be seen as a network of neurons organized in three layers: the input layer, the intermediate layer, which contains hidden nodes, and the output layer. In comparison with linear regression models (such as OLS), the presence of an intermediate layer provides non-linear analysis. (DOCX 62 kb)

Additional file 3: Section Mathematics/formula. (DOCX 15 kb)

Additional file 4: 0, 1, 2 year lag fixed effect regression model. Results of fixed effect regression analysis in extenso. (DOCX 18 kb)

Additional file 5: Trend of spending items and mortality rate. Descriptive analysis of health spending items and mortality rate trend (2011 is identified as the break year). (DOCX 14 kb)

Additional file 6: OLS and MFNN time trend analyses. Results of statistical analyses repeated with alternative health outcome (life expectancy for male and female separately). (DOCX 15 kb)

Additional file 7: OLS Time trend of Male life expectancy. Blue line is real life expectancy trend. Red dots line is predicted life expectancy trend. The 95% CIs are denoted by the grey-coloured area. (DOCX 101 kb)

Additional file 8: OLS Time trend of female life expectancy. Blue line is real life expectancy trend. Red dots line is predicted life expectancy trend. The 95% CIs are denoted by the grey-coloured area (DOCX 103 kb)

Additional file 9: Time trend of DPS using Neural Network model. Blue line is real DPS trend. Red dots line is predicted DPS trend. The 95% CIs are denoted by the grey-coloured area. (DOCX 104 kb)

Additional file 10: Time trend of TAUS using Neural Network model. Blue line is real TAUS trend. Red dots line is predicted TAUS trend. The 95% CIs are denoted by the grey-coloured area. (DOCX 99 kb)

Additional file 11: Time trend of FHE using OLS analysis. Blue line is real FHE trend. Red dots line is predicted FHE trend. The 95% CIs are denoted by the grey-coloured area. (DOCX 102 kb)

Additional file 12: Time trend of FHE using Neural Network model. Blue line is real FHE trend. Red dots line is predicted FHR trend. The 95% CIs are denoted by the grey-coloured area. (DOCX 109 kb)

## Abbreviations

ANN: Artificial Neural Networks
DPS: Directly Provided Services
EU: European Union
FHE: Families’ private Health Spending
GDP: Gross Domestic Product
ISTAT: Italian National Institute of Statistics
MFNN: Multilayer Feed-forward Neural Networks
MR: Mortality Rate
NHS: UK’s National Health Service
OECD: Organization for Economic Co-operation and Development
OLS: Ordinary Least Squares
SSN: Italy’s National Health Service (Servizio Sanitario Nazionale)
TAUS: Total Agreed-Upon Services

## References

[1] Catalano R, Goldman-Mellor S, Saxton K, Margerison-Zilko C, Subbaraman M, LeWinn K (2011). The health effects of economic decline. Annu Rev Public Health.

[2] Karanikolos M, Mladovsky P, Cylus J, Thomson S, Basu S, Stuckler D (2013). Financial crisis, austerity, and health in Europe. Lancet.

[3] Budhdeo S, Watkins J, Atun R, Williams C, Zeltner T, Maruthappu M (2015). Changes in government spending on healthcare and population mortality in the European union, 1995–2010: a cross-sectional ecological study. J R Soc Med.

[4] Ruhm CJ (2015). Recessions, healthy no more?. J Health Econ.

[5] Watkins J, Wulaningsih W, Da Zhou C, Marshall DC, Sylianteng GDC, Dela Rosa PG (2017). Effects of health and social care spending constraints on mortality in England: a time trend analysis. BMJ Open.

[6] Bokhari FAS, Gai Y, Gottret P (2007). Government health expenditures and health outcomes. Health Econ.

[7] Stuckler D, Basu S, McKee M (2010). Budget crises, health, and social welfare programmes. BMJ.

[8] Liaropoulos L, Goranitis I. Health care financing and the sustainability of health systems. Int J Equity Health. 2015;14(1):–80. Available from: http://www.equityhealthj.com/content/14/1/80 10.1186/s12939-015-0208-5 PMC4570753 26369417

[9] Reeves A, McKee M, Basu S, Stuckler D (2014). The political economy of austerity and healthcare: Cross-national analysis of expenditure changes in 27 European nations 1995–2011. Health Policy (New York).

[10] Parmar D, Stavropoulou C, Ioannidis JPA (2016). Health outcomes during the 2008 financial crisis in Europe: systematic literature review. BMJ.

[11] Mladovsky P, Srivastava D, Cylus J, Karanikolos M, Evetovits T, Thomson S (2012). Health policy responses to the financial crisis in Europe.

[12] Eurostat. Social protection 2015. Almost one-third of EU GDP spent on social protection. Vol. 188, Eurostat Newsrealease. 2017 [cited 2018 Jan 5]. Available from: http://ec.europa.eu/eurostat/statistics-explained/index.php/Social_protection_statistics

[13] OECD Reviews of Health Care Quality: Italy 2014 [Internet]. OECD Publishing; 2015. (OECD Reviews of Health Care Quality). Available from: http://www.oecd-ilibrary.org/social-issues-migration-health/oecd-reviews-of-health-care-quality-italy-2014_9789264225428-en

[14] Vercelli M, Lillini R, Quaglia A, Micale RT, La Maestra S, De Flora S (2014). Age-related mortality trends in Italy from 1901 to 2008. PLoS One.

[15] Ferré F, de Belvis AG, Valerio L, Longhi S, Lazzari A, Fattore G, Ricciardi W MA. Italy: Health System Review. Health Systems in Transition, 2014. 2014. Available from: http://www.euro.who.int/__data/assets/pdf_file/0003/263253/HiT-Italy.pdf?ua=1 25471543

[16] OECD (2015). OECD Reviews of Health Care Quality: Italy 2014: Raising Standards.

[17] Italian Ministry of Economy. Il monitoraggio della spesa sanitaria. 2015. Available from: http://www.rgs.mef.gov.it/_Documenti/VERSIONE-I/Attivit%2D%2Di/Spesa-soci/Attivit-monitoraggio-RGS/2015/IMDSS-RS02_15_09_2015.pdf

[18] OECD. OECD Health Statistics. OECD. 2016 [cited 2018 Jan 5]. Available from: http://www.oecd.org/els/health-systems/health-data.htm

[19] OECD (2017). OECD data. Elderly population (Indicator).

[20] Becker DJ, Arora T, Kilgore ML, Curtis JR, Delzell E, Saag KG (2014). Trends in the utilization and outcomes of Medicare patients hospitalized for hip fracture, 2000-2008. J Aging Health.

[21] Yao N, Foltz SM, Odisho AY, Wheeler DC (2015). Geographic analysis of urologist density and prostate cancer mortality in the United States. PLoS One.

[22] Muennig P, Cohen AK, Palmer A, Zhu W (2013). The relationship between five different measures of structural social capital, medical examination outcomes, and mortality. Soc Sci Med.

[23] Shi H-Y, Hwang S-L, Lee K-T, Lin C-L (2013). In-hospital mortality after traumatic brain injury surgery: a nationwide population-based comparison of mortality predictors used in artificial neural network and logistic regression models. J Neurosurg.

[24] Karthikesalingam A, Attallah O, Ma X, Bahia SS, Thompson L, Vidal-Diez A (2015). An artificial neural network stratifies the risks of Reintervention and mortality after endovascular aneurysm repair; a retrospective observational study. PLoS One.

[25] Chiu H-C, Ho T-W, Lee K-T, Chen H-Y, Ho W-H. Mortality predicted accuracy for hepatocellular carcinoma patients with hepatic resection using artificial neural network. Sci World J. 2013:201976. Available from: http://www.ncbi.nlm.nih.gov/pubmed/23737707. 10.1155/2013/201976 PMC3659648 23737707

[26] ISTAT (2017). Health for All. ISTAT.

[27] OECD (2011). A System of Health Accounts.

[28] ISTAT (2015). Calcolo delle rivalutazioni monetarie. ISTAT.

[29] Longo F, Ricci A, Armeni P, Petracca F, Furnari A (2017). Rapporto oasi.

[30] Cantarero-Prieto D, Pascual-Sáez M, Gonzalez-Prieto N (2017). Effect of having private health insurance on the use of health care services: the case of Spain. BMC Health Serv Res.

[31] ISTAT. Il sistema della protezione sociale e le sfide generazionali. Vol. Capitolo 5, ISTAT. 2016 [cited 2018 Jan 5]. Available from: http://www.istat.it/it/files/2016/04/Cap_5_Ra2016.pdf

[32] Armeni P, Cantù E, Carbone C, Longo F, Petracca F, Sommariva S (2015). Italiano Rapporto Oasi 2015. Executive Summary.

[33] Pasini A (2015). Artificial neural networks for small dataset analysis. J Thorac Dis.

[34] Hyndman RJ, Athanasopoulos G. Forecasting: principles and practice. 2015. OText Edition, 2nd Ed. Available from: https://otexts.org/fpp2/.

[35] Sarmiento C (2004). Modelling firm heterogeneity with spatial “trends”. Appl Econ Lett.

[36] Robert L, Harberger C (1969). Labour-Capital Substitution in US manufacturing. The taxation of Income from Capital.

[37] Schwartz S (1994). The fallacy of the ecological fallacy: the potential misuse of a concept and the consequences. Am J Public Health.

[38] Kourentzes N. Can neural networks predict trended time series? 2018 [cited 2018 Jan 5]. Available from: http://kourentzes.com/forecasting/2016/12/28/can-neural-networks-predict-trended-time-series/

[39] Golinelli D, Toscano F, Bucci A, Lenzi J, Fantini MP, Nante N (2017). Health expenditure and all-cause mortality in the “galaxy” of Italian regional healthcare systems: a 15-year panel data analysis. Appl Health Econ Health Policy.

[40] Lopez-Valcarcel BG, Barber P (2017). Economic Crisis, Austerity Policies, Health and Fairness: Lessons Learned in Spain. Appl Health Econ Health Policy.

