# Supplementary material for: Real and predicted mortality under health spending constraints in Italy: a time trend analysis through artificial neural networks
Source: BMC Health Serv Res. 2018 Aug 29;18:671. doi: 10.1186/s12913-018-3473-3 (PMC6116437; doi:10.1186/s12913-018-3473-3)
Supplement: Supplementary file 1 — Spending and control variables. Variables used in time trend analysis and fixed effect regression models. (DOCX 14 kb) [file 12913_2018_3473_MOESM1_ESM.docx]

Additional file 1. Spending and control variables. Variables used in time trend analysis and fixed effect regression models.

| **Expenditure variables** | **DPS: Directly Provided Services** | Spending for social transfers in kind representing all the individual health-related goods and services (OECD glossary of statistical terms) provided free of charge – or with small co-payments – directly by the Italian National Healthcare System. PPP adjusted |
| --- | --- | --- |
|  | **TAUS: Total Agreed-Upon Services** | Spending for Total Agreed-Upon Services: services funded by the Italian SSN, and supplied throughout private healthcare providers. PPP adjusted |
|  | **FHE: Families’ Healthcare Expenditure** | Per capita private healthcare spending of the Italian families. PPP adjusted |
| **Social Condition** | **Unemployment** | Unemployment rate of people aged 15 or more (%) |
|  | **Educational level** | People with at least 8 years of scholastic education (%) |
|  | **GDP** | Per capita regional Gross Domestic Product. PPP adjusted |
| **Lifestyle** | **Obesity rate** | Obese people aged 18 or more (%) |
|  | **Smoke rate** | Smokers aged 15 or more (%) |
|  | **Sport active people** | People who declare practicing physical activity continuously (%) |
| **Healthcare** | **HB: Hospital Beds** | Number of hospital beds per 10,000 population. |
|  | **NN: Number of Nurses.** | Number of nurses employed by the SSN per 10,000 population. |
