# Supplementary material for: Real and predicted mortality under health spending constraints in Italy: a time trend analysis through artificial neural networks
Source: BMC Health Serv Res. 2018 Aug 29;18:671. doi: 10.1186/s12913-018-3473-3 (PMC6116437; doi:10.1186/s12913-018-3473-3)

Additional file 2. Multilayer Feed-forward Neural Networks model. A neural network model can be seen as a network of neurons organized in three layers: the input layer, the intermediate layer, which contains hidden nodes, and the output layer. In comparison with linear regression models (such as OLS), the presence of an intermediate layer provides non-linear analysis.


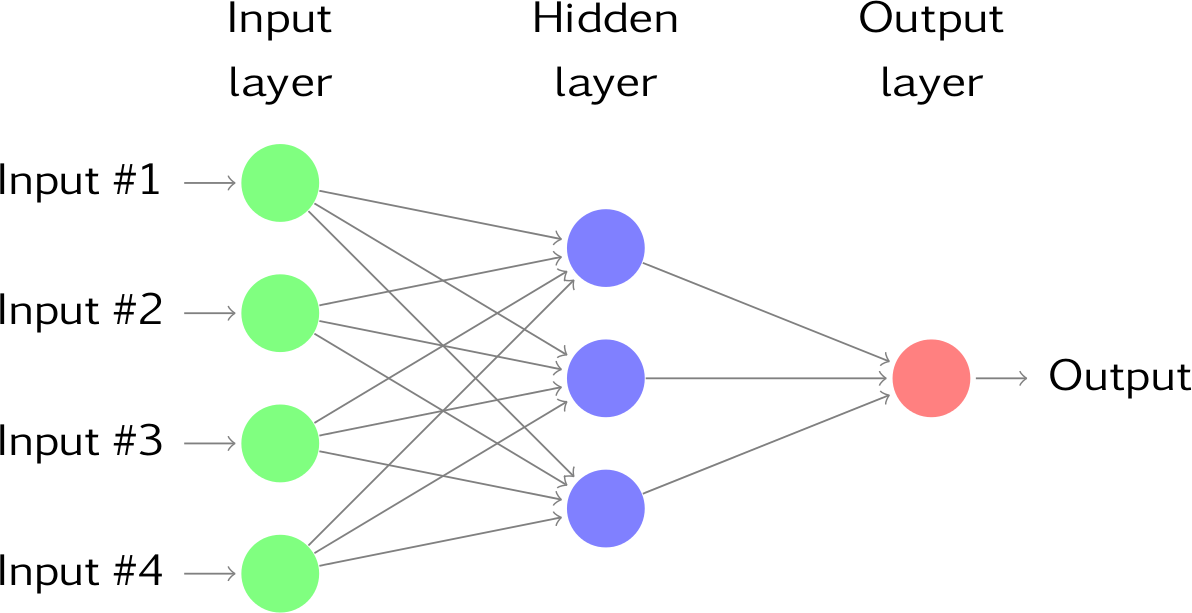

Supplement: Supplementary file 2 — Multilayer Feed-forward Neural Networks model. A neural network model can be seen as a network of neurons organized in three layers: the input layer, the intermediate layer, which contains hidden nodes, and the output layer. In comparison with linear regression models (such as OLS), the presence of an intermediate layer provides non-linear analysis. (DOCX 62 kb) [file 12913_2018_3473_MOESM2_ESM.docx]
