# Supplementary material for: Real and predicted mortality under health spending constraints in Italy: a time trend analysis through artificial neural networks
Source: BMC Health Serv Res. 2018 Aug 29;18:671. doi: 10.1186/s12913-018-3473-3 (PMC6116437; doi:10.1186/s12913-018-3473-3)
Supplement: Supplementary file 3 — Section Mathematics/formula. (DOCX 15 kb) [file 12913_2018_3473_MOESM3_ESM.docx]

**Additional file 3. Section Mathematics/formula**

**Multilayer Feed-forward Neural Networks model:** Following the neural network in Additional file 2, the inputs into the *j*-th hidden neuron are linearly combined to give

$$\theta_{j}= \beta_{j}+ \sum_{i=1}^{4} w_{i,j}x_{i}$$

Where $\beta_{j}$ and the “weights” $w_{i,j}$ are learned from the data and $x_{i}$ is the vector of exogenous variables (i.e. the regressors). In the hidden layer, the result is transformed using a non-linear function, such as a sigmoid/logistic function

$s\left( \theta\right)= \frac{1}{1+ e^{-\theta}}$ ,

to give the input for the next layer, in order to reduce the effect of outliers.

When managing time series, lagged values of the time series can be included in the neural network as inputs. In this paper, we implemented a neural network auto-regression (i.e. a neural network that depends on exogenous values and dependent variable lagged values), NNAR(*p*,*k*), where *p* denotes the lagged inputs and *k* is equal to the number of nodes in the hidden layer. The nnetar() function from the package forecast in R allows to fit a feed-forward neural network, specifying the number of lags and hidden nodes. Since we rely on a single lag of the dependent variable and two nodes in the hidden layer, our predictive model is specified as a NNAR(1,2).

**Fixed effect regression model:** The fixed effect regression model can be defined as follow:

$${MR}_{i,t}= \eta_{i}+ \gamma{HE}_{i,t}+ \delta X_{i,t}+ \theta T+ \varepsilon_{i,t}$$

where $\gamma$ is the vector of regression coefficients for expenditure variables (HE), $\delta$ is the vector of regression coefficients for the control variables X (which vary across the models), $\theta$ is the regression coefficient for the time trend T, while $\eta_{i}$ is the unknown intercept for each of the 20 Italian Regions and $\varepsilon_{i,t}$ is the error term.
