# Supplementary material for: Real and predicted mortality under health spending constraints in Italy: a time trend analysis through artificial neural networks
Source: BMC Health Serv Res. 2018 Aug 29;18:671. doi: 10.1186/s12913-018-3473-3 (PMC6116437; doi:10.1186/s12913-018-3473-3)
Supplement: Supplementary file 4 — 0, 1, 2 year lag fixed effect regression model. Results of fixed effect regression analysis in extenso. (DOCX 18 kb) [file 12913_2018_3473_MOESM4_ESM.docx]

Additional file 4. 0, 1, 2 year lag fixed effect regression model. Results of fixed effect regression analysis in extenso.

Fixed-effects, using 360 observations

Included 20 cross-sectional units

Time-series length = 18

Dependent variable: MR

Robust (HAC) standard errors

|  | ***Coefficient*** | ***Std. Error*** | ***t-ratio*** | ***p-value*** |  |
| --- | --- | --- | --- | --- | --- |
| const | 124.196 | 4.99931 | 24.8426 | <0.0001 | *** |
| FHE_ppp | −0.00336599 | 0.00232751 | −1.4462 | 0.1644 |  |
| FHE_ppp_1 | 0.00483363 | 0.00464409 | 1.0408 | 0.3110 |  |
| FHE_ppp_2 | −0.00668456 | 0.00357951 | −1.8675 | 0.0773 | * |
| time | −1.65774 | 0.130634 | −12.6900 | <0.0001 | *** |
| DPS_ppp | −0.0119611 | 0.00306785 | −3.8989 | 0.0010 | *** |
| DPS_ppp_1 | 0.00484204 | 0.00359409 | 1.3472 | 0.1938 |  |
| DPS_ppp_2 | −0.00156154 | 0.0027642 | −0.5649 | 0.5787 |  |
| TAUS | −0.00544335 | 0.0047467 | −1.1468 | 0.2657 |  |
| TAUS_1 | 0.00117388 | 0.00410547 | 0.2859 | 0.7780 |  |
| TAUS_2 | 0.00441537 | 0.0039919 | 1.1061 | 0.2825 |  |

| Mean dependent var | 93.05188 |  | S.D. dependent var | 11.04208 |
| --- | --- | --- | --- | --- |
| Sum squared resid | 1992.804 |  | S.E. of regression | 2.457397 |
| LSDV R-squared | 0.954473 |  | Within R-squared | 0.944019 |
| Log-likelihood | −818.8328 |  | Akaike criterion | 1697.666 |
| Schwarz criterion | 1814.249 |  | Hannan-Quinn | 1744.021 |
| rho | 0.202001 |  | Durbin-Watson | 1.530114 |

Joint test on named regressors -

Test statistic: F(10, 19) = 606.075

with p-value = P(F(10, 19) > 606.075) = 1.33428e-021

Robust test for differing group intercepts -

Null hypothesis: The groups have a common intercept

Test statistic: Welch F(19, 125.2) = 46.6587

with p-value = P(F(19, 125.2) > 46.6587) = 1.50231e-047
