# Supplementary material for: Real and predicted mortality under health spending constraints in Italy: a time trend analysis through artificial neural networks
Source: BMC Health Serv Res. 2018 Aug 29;18:671. doi: 10.1186/s12913-018-3473-3 (PMC6116437; doi:10.1186/s12913-018-3473-3)
Supplement: Supplementary file 5 — Trend of spending items and mortality rate. Descriptive analysis of health spending items and mortality rate trend (2011 is identified as the break year). (DOCX 14 kb) [file 12913_2018_3473_MOESM5_ESM.docx]

Additional file 5. Trend of spending items and mortality rate. Descriptive analysis of health spending items and mortality rate trend (2011 is identified as the break year)

|  | **DPS** | **TAUS** | **FHE** | **MR** |
| --- | --- | --- | --- | --- |
| Mean 1995-2010 | 3,40% | 3,41% | 1,45% | -2,04% |
| Mean 2011-2014 | -1,45% | -2,71% | 0,12% | -1,69% |
| Difference | -4,85% | -6,13% | -1,33% | 0,35% |
