# Supplementary material for: Real and predicted mortality under health spending constraints in Italy: a time trend analysis through artificial neural networks
Source: BMC Health Serv Res. 2018 Aug 29;18:671. doi: 10.1186/s12913-018-3473-3 (PMC6116437; doi:10.1186/s12913-018-3473-3)
Supplement: Supplementary file 6 — OLS and MFNN time trend analyses. Results of statistical analyses repeated with alternative health outcome (life expectancy for male and female separately). (DOCX 15 kb) [file 12913_2018_3473_MOESM6_ESM.docx]

Additional file 6. OLS and MFNN time trend analyses. Results of statistical analyses repeated with alternative health outcome (life expectancy for male and female separately).

| **Life Expectancy Male** | | | | | |
| --- | --- | --- | --- | --- | --- |
|  |  | **OLS** | | **Neural Network** | |
| **Year** | **Real** | **Prediction** | **95% interval** | **Prediction** | **95% interval** |
| 2011 | 79.47 | 79.89 | (79.47, 80.31) | 79.49 | (78.63, 80.35) |
| 2012 | 79.57 | 80.21 | (79.78, 80.64) | 79.66 | (78.79, 80.54) |
| 2013 | 79.81 | 80.53 | (80.09, 80.97) | 79.79 | (78.93, 80.66) |
| 2014 | 80.28 | 80.85 | (80.40, 81.30) | 79.91 | (79.05, 80.77) |
| **Life Expectancy Female** | | | | | |
|  |  | **OLS** | | **Neural Network** | |
| **Year** | **Real** | **Prediction** | **95% interval** | **Prediction** | **95% interval** |
| 2011 | 84.38 | 84.94 | (84.39, 85.48) | 84.36 | (83.47, 85.24) |
| 2012 | 84.41 | 85.17 | (84.61, 85.73) | 85.84 | (83.53, 86.16) |
| 2013 | 84.62 | 85.41 | (84.84, 85.99) | 84.63 | (83.08, 86.18) |
| 2014 | 84.99 | 85.65 | (85.06, 86.24) | 85.26 | (83.46, 87.06) |
