# Supplementary material for: Real and predicted mortality under health spending constraints in Italy: a time trend analysis through artificial neural networks
Source: BMC Health Serv Res. 2018 Aug 29;18:671. doi: 10.1186/s12913-018-3473-3 (PMC6116437; doi:10.1186/s12913-018-3473-3)

Additional file 7. OLS Time trend of Male life expectancy. Blue line is real life expectancy trend. Red dots line is predicted life expectancy trend. The 95% CIs are denoted by the grey-coloured area


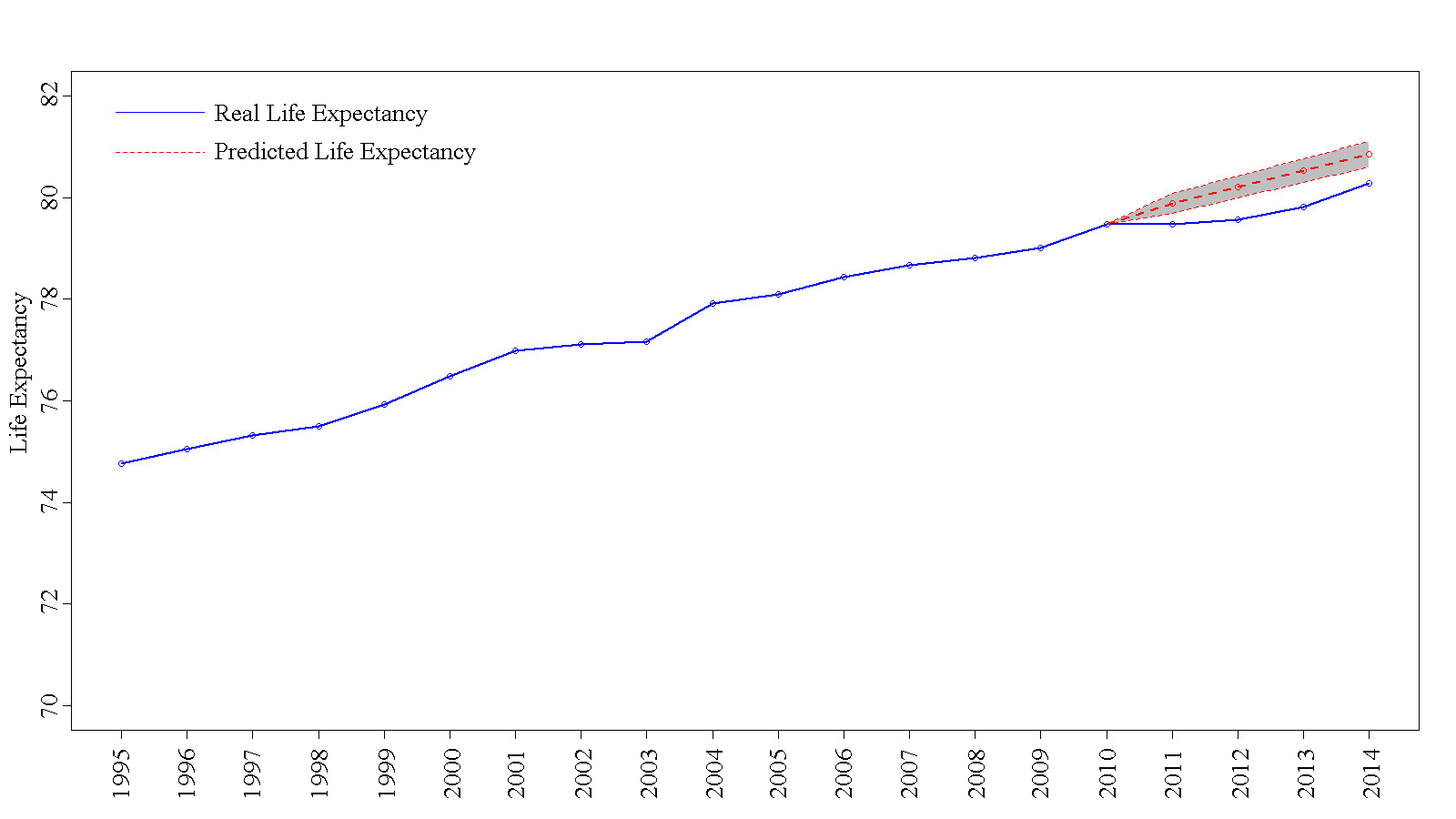

Supplement: Supplementary file 7 — OLS Time trend of Male life expectancy. Blue line is real life expectancy trend. Red dots line is predicted life expectancy trend. The 95% CIs are denoted by the grey-coloured area. (DOCX 101 kb) [file 12913_2018_3473_MOESM7_ESM.docx]
