# Supplementary material for: Real and predicted mortality under health spending constraints in Italy: a time trend analysis through artificial neural networks
Source: BMC Health Serv Res. 2018 Aug 29;18:671. doi: 10.1186/s12913-018-3473-3 (PMC6116437; doi:10.1186/s12913-018-3473-3)

Additional file 12. Time trend of FHE using Neural Network model. Blue line is real FHE trend. Red dots line is predicted FHR trend. The 95% CIs are denoted by the grey-coloured area.


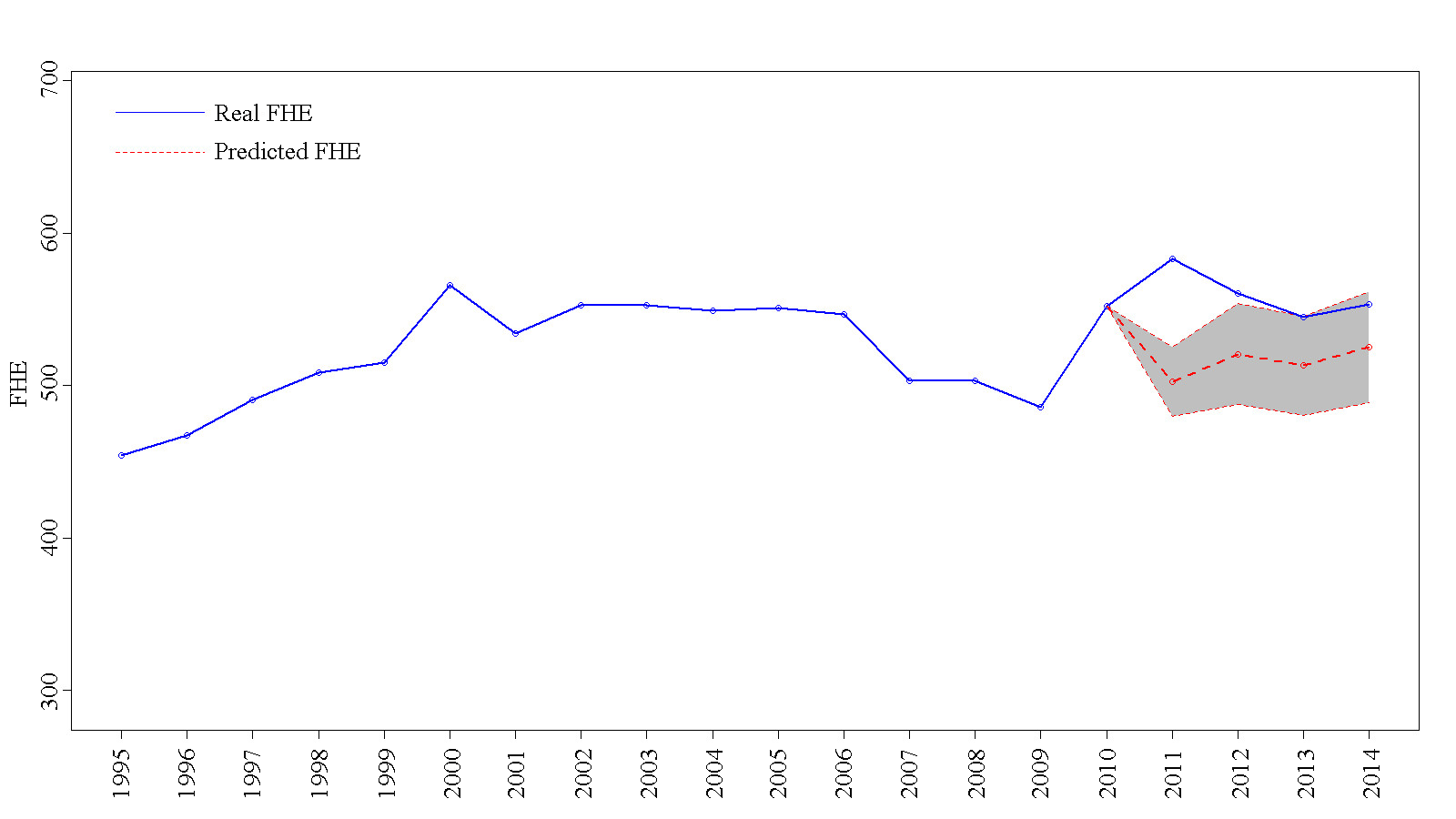

Supplement: Supplementary file 12 — Time trend of FHE using Neural Network model. Blue line is real FHE trend. Red dots line is predicted FHR trend. The 95% CIs are denoted by the grey-coloured area. (DOCX 109 kb) [file 12913_2018_3473_MOESM12_ESM.docx]
